# Supplementary figures and images for: Crossing complexity of space-filling curves reveals entanglement of S-phase DNA
Source: PLoS One. 2020 Aug 31;15(8):e0238322. doi: 10.1371/journal.pone.0238322 (PMC7458320; doi:10.1371/journal.pone.0238322)

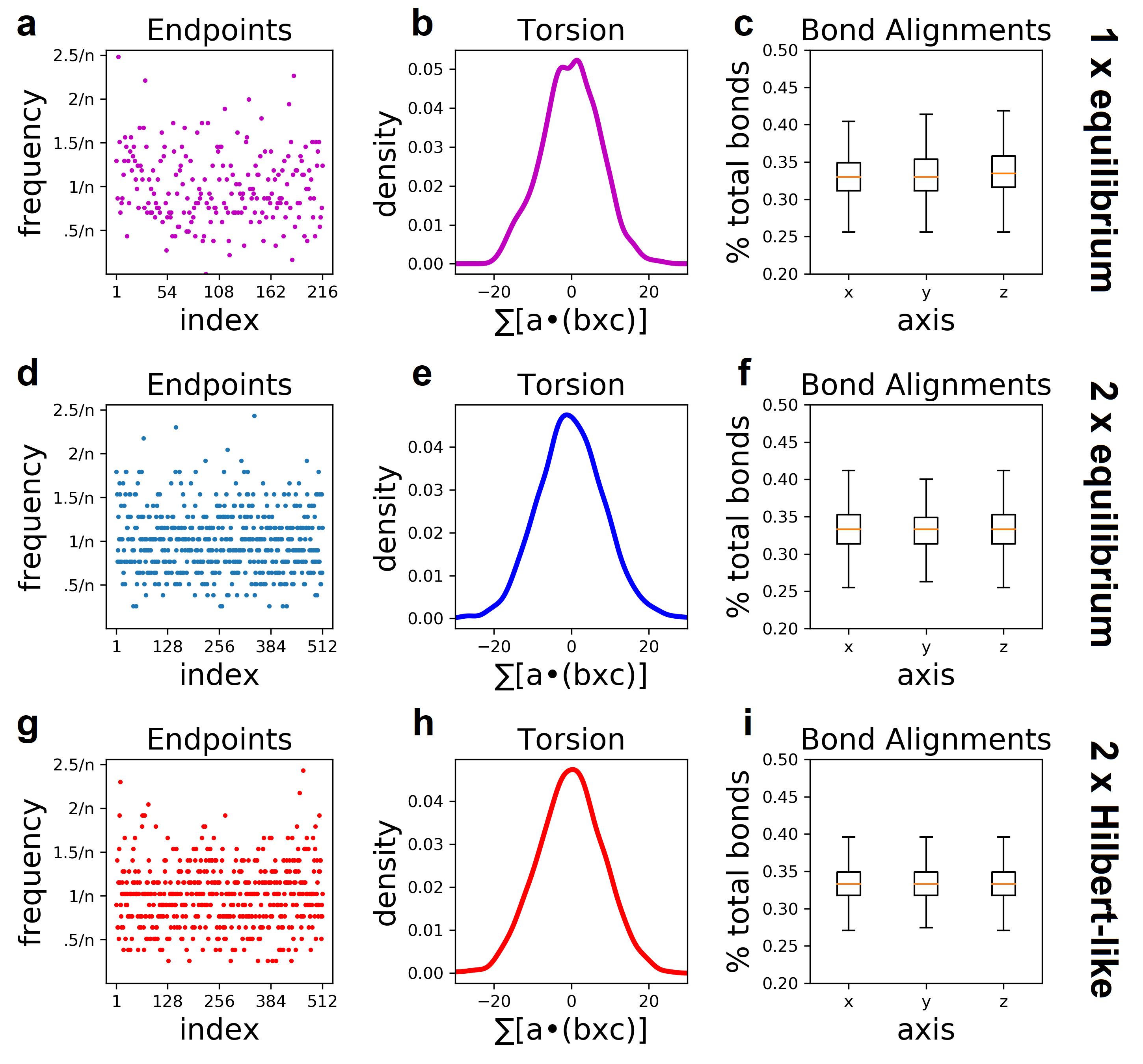

Supplement: S1 Fig — (PNG) [file pone.0238322.s003.png]
