# Supplementary material for: Crossing complexity of space-filling curves reveals entanglement of S-phase DNA
Source: PLoS One. 2020 Aug 31;15(8):e0238322. doi: 10.1371/journal.pone.0238322 (PMC7458320; doi:10.1371/journal.pone.0238322)
Supplement: Attachment — Submitted filename: Response to Reviewers.pdf [file pone.0238322.s004.pdf]

Edward Via College of Osteopathic Medicine
VIRGINIA • CAROLINAS
AUBURN

Primary Care Research Network
Center for Bioinformatics and Genetics
Nicholas Kinney, PhD
2265 Kraft Drive
Blacksburg, Virginia 24060
Phone: 301.338.1181
Email: nkinney@vcom.vt.edu

July 25, 2020

Dr. Miguel Angel Sanchez Granero
Academic Editor of PLOS ONE
RE: POINT BY POINT RESPONSE TO REVIEWERS

Dear Dr. Granero:

In the pages that follow we respond point-by-point to each of the reviewer comments concerning our manuscript: "**CROSSING COMPLEXITY OF SPACE FILLING CURVES REVEALS ENTANGLEMENT OF S-PHASE DNA**". The manuscript is a substantial revision that address several major concerns. Both reviewers were concerned about the lack of quantitative results regarding knot complexity. The revised manuscript de-emphasizes the topic of knots, knot complexity, and Alexander polynomials. The motivation for the manuscript is to learn more about the statistical properties of crossing complexity for different classes of space filling curves. The shift in tone streamlines the manuscript. The overall message of the manuscript was unclear for second reviewer. This is a serious issue and we have updated appropriate passages in the abstract, introduction, and conclusion to provide further clarity. Reviewer one requested that we rationalize our data with a geometric argument. This led to fruitful new results and a new section in the manuscript. The numerous additional revisions are summarized in the pages that follow.

We have updated a statement in the manuscript that clarifies the study's funding: "The authors received no specific funding for this work."

We are excited to share our revised manuscript with the peer reviewers and look forward to hearing back. There are no related articles published or under consideration. If there are any specific questions that need a conversation, my cell is 301 338-1181.

Sincerely,

Nicholas Kinney

Dr. Nicholas Kinney
Primary Care Research Network and the Center for Bioinformatics and Genetics, VCOM
Professor of Biomedicine, VCOM

Reviewer #1

While the work has some interesting aspects, some parts lack necessary detail, some other parts may benefit from more insight from the results already reported in the literature. The terminology used in the introduction and abstract for the different curves is a bit confusing. The authors should try to clarify more sharply the distinction of ``fractal", ``space-filling", ``Hamiltonian" and ``equilibrium" curves and their relation to the presence of knots. Firstly, ``fractal" does not necessarily mean the curves don't have knots, but simply that they are self-similar. Ordinary random walk is also fractal, but exhibits knots when computed on properly closed curve [Meyer et al, ACS Macro Lett. 2018, 7, 6, 757--761].

We agree that some of the terminology is too informal. The term "fractal" is too ambiguous and for the most part has been removed from the manuscript in favor of more specific terms such as "Hilbert-like" and equilibrium. The definition of space-filling is now given immediately in the abstract which begins:

> Space-filling curves have been used for decades to study the folding principles of globular proteins, compact polymers, and chromatin. Formally, these are configurations with fractal dimension equal to the embedding space; informally, they correspond to a polymer melt. Although not quite a melt, the folding principles of Human chromatin are likened to the Hilbert curve: a type space-filling curve.

The revised manuscript de-emphasizes the topic of knots, knot complexity, and Alexander polynomials. Knot complexity is still mentioned in the abstract but only as a segue to crossing complexity: the focus of the paper. To avoid confusion knots, knot complexity, and Alexander polynomials are mostly removed from the manuscript except for a few appropriate places in the introduction. It is not our intention to avoid the topic of knots; rather, the focus of the paper is on crossing complexity. Per request of the second reviewer we compute knot determinants for configurations of equilibrium, intermediate, and fractal-like (now called Hilbert-like) configurations (see below); however, those results are not included in the manuscript.

Second, ``space-filling" typically denotes curves with fractal dimension d, i.e. that of the embedding space. In other words, the exponent α (commonly named ν) having the value of $1/d$ where d is the space dimension. Based on the constructions in [Smrek, Grosberg, Physica A 2013, 392 (24), 6375-88], these curves could also be knotted.

The definition of "space-filling" is now given immediately in the abstract and reiterated in the introduction (see our previous response).

Third, connection to ``Hamiltonian" paths i.e. ones visiting every vertex of a graph just once should be made.

A member of our team with a background in graph theory preferred to avoid the term "Hamiltonian". The concern was that some readers may only associate the term with visiting nodes of an undirected or directed graph. In retrospect it is appropriate to define this term so

that the connection is made. A simple change to the introduction now makes the connection clear:

> Given a set of points (x,y,z), a SFC visits each once and only once; consequently, they are Hamiltonian and sometimes referred to as Hamiltonian paths

Fourth, ``equilibrium" curves should be probably discussed from the polymer melt perspective [Meyer et al above].

The link between polymer melts, compact polymers, and space filling curves is now clarified in the introduction; and, we cite the paper recommended by the reviewer. From the fourth paragraph of the introduction:

> Space filing curves, compact polymers, and chromatin are related by a mutual correspondence to the state of polymers in a melt. Broadly speaking, this state corresponds to that of dense intermingled blobs, but, one in which polymers are still in thermal motion. Although chromatin is not quite a melt, such a dense state with the potential to form tangles and knots is a conceivable hazard to chromatin folding and unfolding. However, simulations suggest that knots in melts are fewer than expected; in any case, human chromosomes are probably not in equilibrium. Chromatin is believed to fold into configurations that limit entanglement between and within chromosomes. A model consistent…

Fifth, a brief explanation of what is meant by knots on open curves would help the reader.

The revised manuscript de-emphasizes the topic of knots, knot complexity, and Alexander polynomials (see our first response). The motivation for the manuscript is to learn more about the statistical properties of crossing complexity for different classes of space filling curves. The original manuscript misguided the reader by including statements about knots and knot complexity. Consequently, both reviewers (particularly the second reviewer) anticipated quantitative results comparing knot complexity and crossing complexity. To avoid confusion the revised manuscript mostly removes passages related to knots and knot complexity. So, we do not discuss the subtleties of knots on open curves. This is not to avoid the helpful suggestions of the reviewer; rather, it helps the manuscript and reader focus exclusively on the topic of crossing complexity. Per request of the second reviewer we compute knot determinants for configurations of equilibrium, intermediate, and fractal-like (now called Hilbert-like) configurations (see below); however, those results are not included in the manuscript.

Sixth, another useful paper in the context of chromatin and knottedness is [Goundaroulis et al, Biophysical Journal, 118, 9, 5 2020, 2268--2279]. A deeper exposure of the differences in the chromatin context could be found in [Halverson et al 2014 Rep. Prog. Phys. 77 022601].

We now cite both papers. As stated above, the revised manuscript mostly removes passages related to knots and knot complexity.

The latter reference, besides the exponent α (ν), discusses also exponent β that characterizes fractal dimension of the curve's boundary for the SFCs or the scaling of the number of contacts between two curves. This seems strongly related to the crossing number, which presumably characterizes how intermixed the two curves in question are. It would be useful to discuss the results in this context.

This is indeed an interesting possibility that should be discussed. A thorough quantitative comparison of β and crossing complexity is probably beyond the scope of this work; but, the re-written second discussion paragraph does a better job discussing both metrics in addition to knot complexity. The relationships between these metrics are proposed a future line of inquiry:

> Additional contributions of this work stem from an investigation of crossing complexity for SFCs. Our results are twofold: (a) Hilbert-like configurations limit crossing complexity between chromosomes and (b) Hilbert-like configurations do not limit crossing complexity once doubled akin to S-phase DNA. Despite these contributions, crossing complexity remains understudied compared related metrics such as knot complexity (Δ) and surface smoothness (β). Here surface smoothness refers to the monomers of one curve in contact with other curves governed by the exponent β: $n_{surf} \sim N^{\beta}$. Hilbert-like curves are characterized by β = 2/3, knot complexity Δ(-1) = 1, and modal crossing complexity (see results) approximately zero. It is natural to seek a quantitative comparison of these metrics. We hypothesize the correlation is strongest for crossing complexity and surface smoothness; however, a thorough study is beyond the scope of this work. In anticipation of more widespread use, we parallelize the crossing complexity using open MPI and develop novel visualizations over the space S^2 (spherical surface). Our code is freely available online.

Fig3: It simply looks like there are fewer chain crossings in the face directions in comparison to the corner directions. This is no surprise, as the latter has more sites by a factor of $\sqrt{3}$, which agrees with the scale in (e): $1340 \sqrt{3}=2320$. In this sense it would be useful to normalize the net crossing numbers in a given direction by the length of the separation path in a given direction, and compute the respective distribution only afterwards.

We agree it is necessary to check that our results are robust to this type of normalization. We have added a methods section detailing the normalization procedure and show robustness for one of our key results. The section appears in the updated manuscript and is provided here. Note that crossing complexity without normalization is still used throughout our results. We feel this simplifies the interpretation of crossing complexity for most readers; the results section encourages interested readers to review the new section on normalization.

> A main result of this work hinges on distributions of enumerated chain crossings (crossing complexity) for pairs of equilibrium, intermediate, and fractal-like space filing curves (figure 4a-c). Differences in those distributions suggest that crossing complexity depends on the folding principles specific to each ensemble (figure 4d and 4e). However, chain crossings visualized for one snapshot (figure 3) appear to be anisotropic: relatively few chain crossings are observed in face directions compared to corner directions. We checked robustness of our results by normalizing enumerated crossing numbers by the lattice size measured in the direction of each separation path. The following example provides furthest clarification:

a. Consider a single snapshot consisting of two curves on the same lattice (figure 10a). Chain crossings are enumerated for test directions that evenly cover the space S^2 (figure 10b). Enumerated chain crossings for ensembles of snapshots produce different distributions depending on how two curves are folded (figure 10c and figure 4d).

b. Consider the same chain crossings weighted by the lattice size measured in the direction of each separation path (figure 10d). Dividing by weight produces a new set of normalized chain crossings (figure 10e). Normalized chain crossings for the same ensembles – equilibrium, intermediate, and fractal-like – produce different distributions; however, our conclusions are unchanged (figure 10f). Differences in the distributions suggest that crossing complexity depends on the folding principles specific to each ensemble.

pg10 ln 153: ``fractal curves are easy to pull apart". Not all of them, it likely depends on β. It looks like here by fractal curves the author mean the traditional ones with smooth surface i.e. $\beta=(d-1)/d$

Yes, we mean the class of fractal curves having β=2/3. Throughout the manuscript we now refer to this specific class of fractal curves as Hilbert-like curves as in Smrek and Grosberg, 2015. We also do a better job placing this interpretation in the context of previous results:

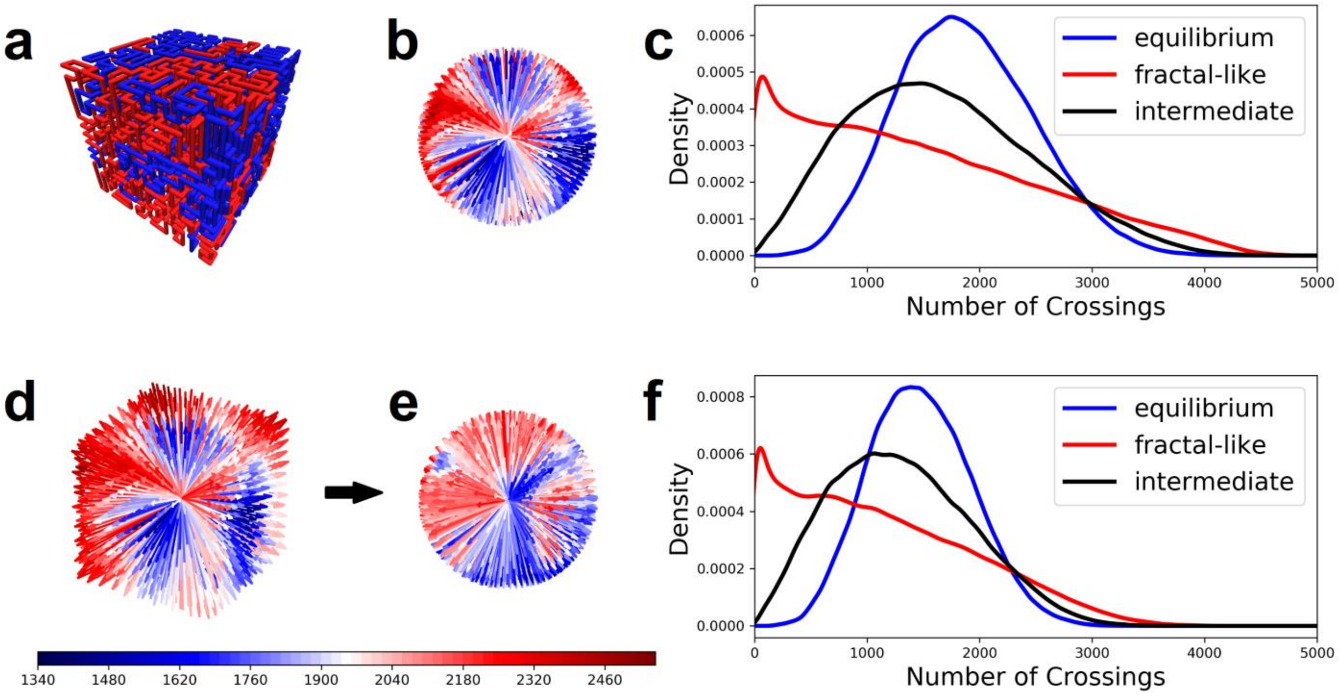

New Figure 10 – a key result of this work is robust to crossing complexity normalization. a, snapshot of two space filling curves on a lattice. b, chain crossings enumerated for a set of test directions. c, the distribution of chain crossings over three different ensembles of configurational snapshots: distributions differ for equilibrium, intermediate and fractal-like curves. d, chain crossings enumerated for a set of test directions and weighed by the lattice size measured in the direction of each separation path. e, chain crossings normalized by weight. f, results are robust to crossing complexity normalization.

> The distribution of enumerated chain crossings clearly differs for each ensemble (figure 4d and 4e). The Hilbert-like ensemble produces an asymmetric (non-normal) distribution with average number of crossings lower than equilibrium and intermediate ensembles, respectively. Roughly speaking, Hilbert-like curves require fewer chain crossings to pull apart; this interpretation is consistent with easy unfolding of fractal-like configurations in simulation (10) and their lack of entanglement inferred from knot complexity (12). See figure S27 of the latter reference.

pg10 ln 156-161: The classification of the curves to the three classes is artificial, because equilibrium class can contain also fractal-like one.

True, fractal-like curves (how called Hilbert-like) are a subset of the equilibrium curves. However, we don't think this makes the classification artificial. We defend the exercise for three reasons:

First, consider the number of Hilbert-like curves compared to equilibrium curves on a 4x4x4 lattice. From Schram, 2013 we know there are 2.8e16 equilibrium curves. From Smrek and Grosberg 2015 we know there are 7.0e9 (x12) Hilbert like curves. Thus, Hilbert-like curves are less than 1 in a million on the 4x4x4 lattice; even less on the 16x16x16 lattice considered in our work. Consequently, Hilbert-like curves contribute very little to the crossing complexity distribution for equilibrium curves (blue line in figure 4d of our manuscript)

Second, the classification exercise is not perfect. There are indeed a few equilibrium curves that – statistically speaking – have Hilbert-like crossing complexity (figure 4f of the manuscript). In retrospect, we think the point of the classification exercise is to consider individual snapshots in addition to statistics of the entire ensemble. Essentially, the snapshot variability (representative of cell-cell variability) should not nullify analysis of the ensembles. The updated motivation appears in our revised manuscript:

> In addition to ensemble differences, links to chromatin folding are bolstered by considering individual snapshots (loosely representative of individual cell nuclei). In particular, is crossing complexity sufficient to discriminates equilibrium, intermediate, and fractal-like configurational snapshots? Or simply, what is the snapshot (cell) variability?

pg10 ln164 -165: ``Physical interpretation of the crossing complexity is that chains are easiest to pull apart in directions with few accumulated crossings (figure 3)". Is this a result of a simulation, experiment or just an intuitive idea? This should be made clear.

We agree and think that entire paragraph should be clarified. The revised version is included here:

> Crossing complexity is also relevant for states of DNA that arise in S-phase after replication. First principles suggest this state consists of two strands (template and newly synthesized) juxtaposed in register. In subsequent phases the two strands separate. We hypothesize that the configuration of replicated S-phase DNA influences the number of crossings needed for strand separation. We test this hypothesis in two steps. First, we construct snapshots of S-phase DNA (described below). Then we compare crossing complexity for equilibrium, intermediate, and fractal-like ensembles. The results – which to not support our hypothesis – lead to one of the main conclusions

of this study: the crossing complexity of doubled space filling curves is the same regardless of how they are folded.

On the s-phase model: There should be a geometric argument why the doubled curves show the same crossing number distributions.

Great idea! We have added a new section to the results of our manuscript that outlines an argument. We also include the argument here:

Surprisingly, the doubled (S-phase) configurations appear to produce the same distribution of chain crossings regardless of their underlying folding principles; equilibrium, intermediate, or fractal-like. We rationalize this observation with a simple geometric argument.

First, consider projecting layers of a space-filling curve in the plane perpendicular to one axis; for example, the space filling curve in figure 6a and its projections in figure 6b-e. In this case, bonds parallel to each projection number 11, 9, 10, and 11, respectively (figure 6b-e). In fact, the parallel bonds in each projection are predictable. Simply put, two thirds of the 63 total bonds are divided into four layers. Therefore – assuming no spatial anisotropy – we expect 10-11 bonds in each projection regardless of folding principles.

Next, consider the doubled curve (red figure 6f). Follow a single bond through each layer (red in figure 6g-j). The probably of chain crossing is proportional to the number of bonds in each layer. Regardless of how the blue curve is folded, each layer has 24 positions for 10-11 bonds. Consequently, we expect the red segment to produce ~2 chain crossings as it traverses layers of the blue curve (figure 6g and 6j). Crossing complexity simply sums over each segment, therefore, is not expected to depend on folding principles for the doubled curves.

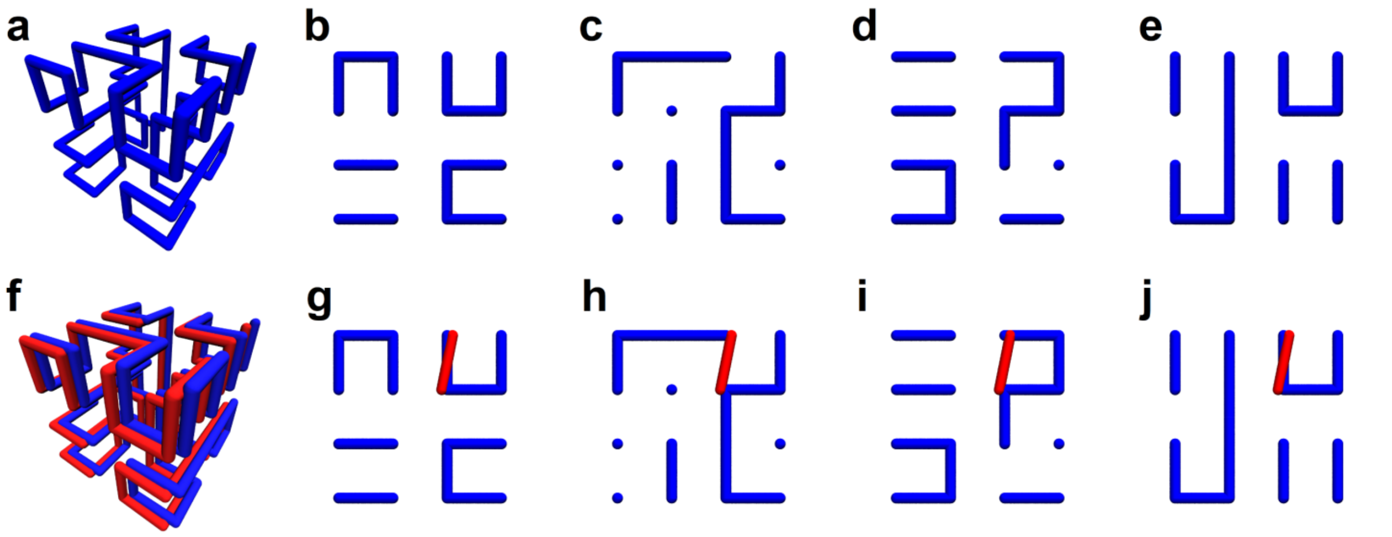

New Figure 6 – a simple geometric argument suggests that doubled space-filling curves produce the same distribution of enumerated chain crossings regardless of folding principles. a, space filling curve on a 4x4x4 lattice. b-e, four layers of the space filling curve projected along one axis. f, a doubled 4x4x4 space filling curve. g-j, a single segment of the doubled curve followed through each layer of the blue curve. Chain crossings occur in panels g and j.

What if we follow the red segment in skew directions; i.e. directions not perpendicular to the plane of the blue curve? Even in these cases (which are the majority) our argument remains fundamentally unchanged. Bonds in the blue curve are sufficiently random that the probability of chain crossing for each red segment is the same regardless of folding principles.

On the ergodicity discussion. It is stated correctly that the indications suggest, but do not prove unbiased sampling. Would it be possible to test this on smaller cases where the total enumeration is possible? "Small" Hamiltonian paths are enumerated in [Schram and Schiessel, 2013 J. Phys. A: Math. Theor. 46 485001] while some smooth fractal curves in [Smrek and Grosberg 2015 J. Phys. A: Math. Theor. 48 195001].

We thank the reviewer for drawing our attention to two important papers on the enumeration of Hamiltonian walks and self-similar space filling curves, respectively. Both are remarkable achievements! To give credit to these works we make a small but significant change to the first paragraph of the introduction; previously we stated:

> “the number of solutions is virtually infinite even for modest size lattices (1)”, citing Jacobsen 2007

Our revised manuscript reads:

> “the partition function scaling grows exponentially with curve length (L): $Z \sim 2.135^L$ for equilibrium curves (1) and $Z \sim 1.35699^L$ for Hilbert like curves (2)” citing Schiessel 2013; and, Smrek and Grosberg 2015

The aforementioned papers are now the first and second cited in our manuscript, respectively. We agree that exact enumeration on small lattices is the best way to have confidence in ergodicity of our approach; however, this is likely outside the scope of our study for one simple reason. Specifically, most of our configurational snapshots place two (equal length) space filling curves on the same lattice. Partition function scaling probably grows even faster for these configurations compared to just one space filling curve. In fact, some work has been done to enumerate multiple space filling curves on a lattice: *Exact enumeration of Hamiltonian circuits, walks, and chains in two and three dimensions, Jacobsen 2007. Even though the Jacobsen results are not limited to equal size curves – as in our case – we can* still postulate that two equal size curves on a single lattice scale faster than one:

- **First, consider Hamiltonian path configurations for the 3x3x4 lattice; results from Jacobsen show they number 677,849,536.**
- **Next, consider Hamiltonian path configurations for the 2x3x3 lattice; results from Jacobsen show they number 16,880.**
- **finally consider placing two Hamiltonian paths of equal length on the same 3x3x4 lattice. Do this by dividing the lattice into 2x3x3 halves; one for each Hamiltonian path. The number of configurations for both halves combined is $16{,}880^2$ = 284,934,400. Even though this is an obvious underestimate, it is little less than half of 677,849,536: the number of configurations for one curve on the 3x3x4 lattice. We have not even considered states where the two curves mix which will considerably increase the number of configurations.**

It stands to reason that 2 curves on a 4x4x4 lattice will number more than the figure (2.8e16) given by Schram, 2013. Thus, even exact enumeration on the 4x4x4 lattice may be unfeasible for the current study. We could conceivably preform exact enumeration on smaller 3x3x3 or 3x3x4 lattices; however, odd numbered lattice sometimes have problems of parity which could

mislead our conclusions. Suffice it to say, exact enumeration is outside the scope of the current study, we may pursue the idea in a separate study; for example, by beginning with ~36,000 elementary cubes in Schiessel 2013.

Finally, we would like to reiterate a result from our manuscript and provide the reviewer a figure prepared for the supplementary material. We compute statistics for an ensemble of 1000 uncorrelated configurational snapshots; each snapshot is generated on an 8x8x8 lattice with two chains; i.e. each chain occupies 256 points. Our results are summarized in figure R3d-f below (figure 7a-c from the manuscript). We compare these results to snapshots of single chains on a 6x6x6 lattice. Here, each chain occupies 216 points which is comparable to the 256 points occupied by each chain in the 8x8x8 configurations. Results for the single chain 6x6x6 configurations (figure R3a-c below) are similar to the double chain 8x8x8 configurations (figure R3d-f below). Importantly, the Mansfield algorithm for single chains likely is ergodic (1) which bolsters confidence in the double chain sampling.

(1) Mansfield, Marc L. "Unbiased sampling of lattice Hamilton path ensembles." *The Journal of chemical physics* 125.15 (2006): 154103.

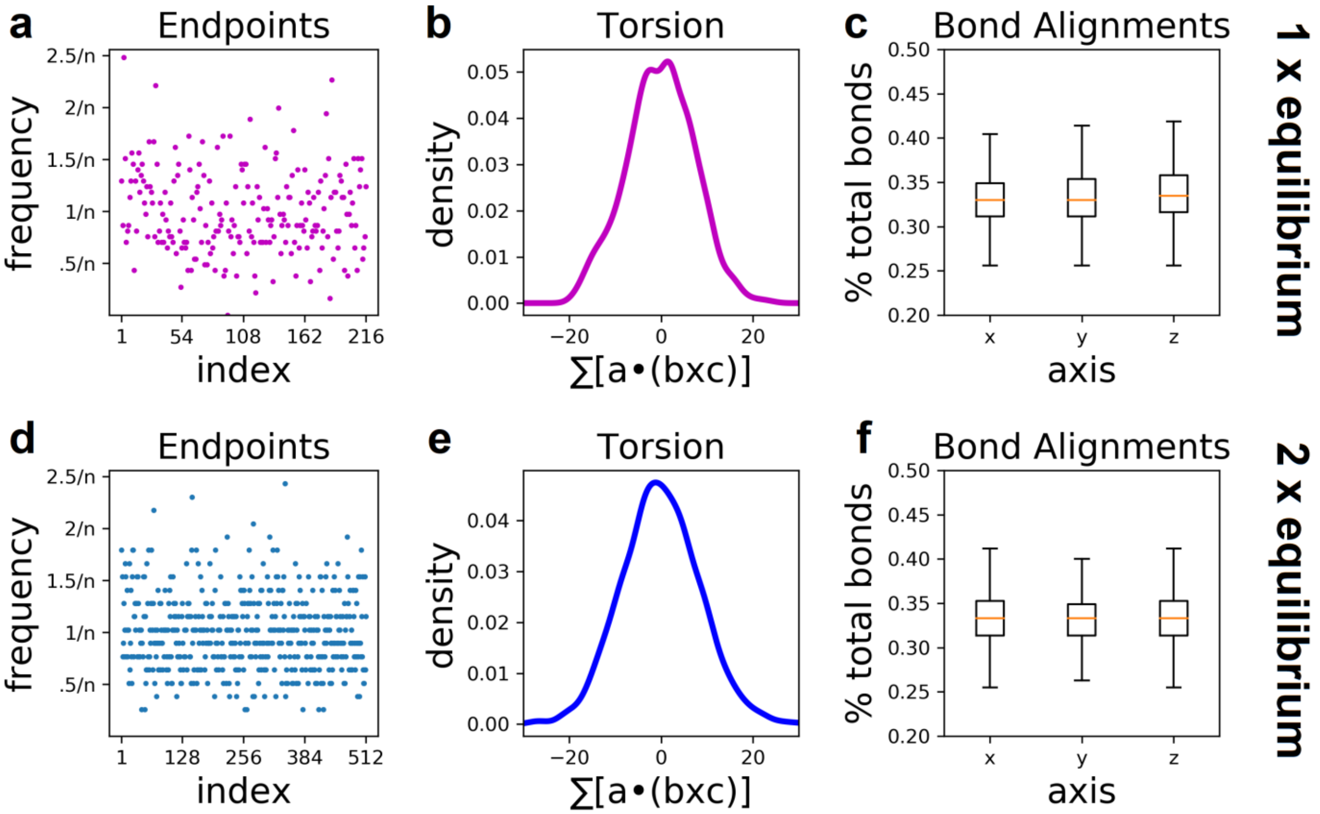

Figure R3 – Statistics of lattice space filling curves suggest they are ergodic. Top row, results for equilibrium configurations of one curve on a 6x6x6 lattice. **Bottom row,** results for equilibrium configurations of two curves on an 8x8x8 lattice. **a and d,** Probability for chain ends at each lattice site are distributed around the expected value: inverse of the lattice size (n). **b and e,** distribution of total chain torsion (measured with the triple product) has zero mean. **c and f,** bonds for each chain have equal probability of alignment along the x, y, and z axis. These findings suggest – but do not prove – that configurations are sampled without bias.

Besides, is it trivially obvious that the endpoints positions of the fractal curves must be uniformly distributed within the cube (pg14 ln229)? From the latter paper this does not seem to be so.

In short, it is not trivially obvious. Just look at 3x3x3 (or any odd numbered lattice) to see issues of parity. The updated manuscript corrects for this inaccuracy. The revised passage in the updated manuscript is included below. We cite the aforementioned paper but also note that chain endpoint occupancy is unclear for the 3rd and 4th order Hilbert-like curves considered in our manuscript; the difficulty is well stated in discussion paragraph 3 of Smrek and Grosberg, 2015. Our revised passage:

> First, we investigate probabilities of chain endpoint occupancy, i.e. how often chain ends occupy each lattice site. We find the probability of chain ends at each lattice site distributed around the inverse of the lattice size (figure 6a). This seems reasonable for an unbiased configuration (it probably is in our case); however, it is not the case for every lattice. For example, the 3x3x3 lattice has well known parity rules that exclude chain endpoints from occupying even numbered (center of each edge) lattice sites. More complicated parity rules have been shown for second order Hilbert curves (2). Thus, it should not be assumed a priori that unbiased configurations produce a normal distribution of endpoints. Simply put, the normal distribution of chain endpoints in our configurations does not obviously rule out ergodicity of the multi-chain algorithm used throughout this work. To have more confidence in the result we compare to an ensemble of single chain configurations on a 6x6x6 lattice (each chain occupies 216 points). Note that the Mansfield algorithm for single chains is very likely ergodic (3). We enumerate 1000 single chain configurations on a 6x6x6 lattice and find a similar normal distribution of chain end occupancy (figure S1).

Another minor change is made when discussing statistics for fractal-like (now properly called Hilbert-like) configurations:

> We reiterate that this does not obviously rule out ergodicity of the algorithm used throughout this work

Correlation times of the curves' generation algorithms are not discussed, only some number is mentioned on ln363. How do we know the samples are uncorrelated?

The revised manuscript provides further clarity for this important point. Correlation times (iterations) as a function of lattice size are given in figure 2 of Mansfield's 2006 paper, *Unbiased sampling of lattice Hamilton path ensembles*. The 16x16x16 lattice requires approximately 10^4 iterations for uncorrelated snapshots. To be safe we begin with 10^5 iterations, an order of magnitude more than the minimum. We added a few sentences for clarification:

> To avoid correlated snapshots, our algorithm begins with 10^5 iterations using a unique random seed. This number is not an arbitrary choice. For 16x16x16 lattices approximately 10^4 iterations are needed to remove correlations (3); thus, we safely exceed the minimum number of iterations by an order of magnitude.

In summary, this is potentially an interesting paper on the statistical properties of sub-classes of Hamiltonian paths, but significant clarification and much more serious connection to the literature is absolutely necessary.

We thank Dr. Grosberg for the constructive feedback and thorough review of our manuscript.

Reviewer #2

The generalization of Mansfield's algorithm is one of the motivations behind the present work, and I think it is an interesting one. Unfortunately, concerning the rest of the paper, I am much less positive and I am now trying to explain why:

Obviously, the main message of this work is that since knottedness is not sufficient to discriminate between equilibrium and fractal curves, chain crossing is proposed as a better indicator (Fig. 4): intuitively, deciding that two compact curves may or may not be knotted (based on some knot invariant) could indeed be complicate so I find reasonable what the authors say. Unfortunately, I would have preferred to see their claim motivated by quantitative analysis, while they only point to an obscure (at least for me!) reference (Ref. [12], author: Golyk VA) which is not available. I find this unfair, references must be available either published or at least in preprint form.

In retrospect, we agree that the Golyk paper is obscure. The manner of its publication is unclear; and, its unclear that it was peer reviewed. To be fair we still cite this paper, but only once.

More importantly though, we have adjusted the message of our manuscript. As written there was too much emphasis on knots and knot complexity. The revised manuscript de-emphasizes the topic of knots, knot complexity, and Alexander polynomials (see also our responses to reviewer one). The motivation for the manuscript is to learn more about the statistical properties of crossing complexity – which is understudied – for different classes of space filling curves. The original manuscript misguided the reader by including too many statements about knots and knot complexity. Consequently, both reviewers anticipated quantitative results comparing knot complexity and crossing complexity. To avoid confusion the revised manuscript mostly removes passages related to knots and knot complexity. The updated motivation and key results are now explicitly clear in the abstract:

> Crossing complexity is an understudied alternative better suited for quantifying entanglement between chromosomes. Do Hilbert-like configurations limit crossing complexity between chromosomes? How does crossing complexity for Hilbert-like configurations compare to equilibrium configurations? To address these questions, we extend the Mansfield algorithm to enable sampling of fractal-like space filling curves on a simple cubic lattice…. Our main results are twofold: (a) Hilbert-like configurations limit entanglement between chromosomes and (b) Hilbert-like configurations do not limit entanglement in a model of S-phase DNA. Our second result is particularly surprising yet easily rationalized with a geometric argument.

The second paragraph of the discussion also reiterates that both results are components of our main message:

> Additional contributions of this work stem from an investigation of crossing complexity for SFCs. The main message is twofold: (a) Hilbert-like configurations limit crossing complexity between chromosomes and (b) Hilbert-like configurations do not limit crossing complexity once doubled akin to S-phase DNA.

Even though passages on the topic of knots are largely removed from the manuscript, we still provide the reviewer with some quantitative analysis below.

But even before my point (1) I have an even more serious concern: I suppose that the polymers chains simulated by Kinney et al. are linear, open polymers. Rigorously, knots exist only for closed curves (rings): yet, knots can still be generalized to open curves provided some numerical "tricks" or definition are adopted (see for instance: Micheletti, Marenduzzo, Orlandini, Phys. Rep. (2011)). Do the authors look into this literature? I think they have to: these "tricks" are relatively standard now, and the authors should analyze their curves based on these methods. Only after that, the comparison to decide which indicator between knottedness and crossing performs better would reveal its true potential.

To reiterate, the manuscript has shifted away from a discussion of knots and knot complexity and aims to focus exclusively on crossing complexity. We cite the recommended paper in an appropriate place in the updated manuscript. Here we provide the reviewer with quantitative analysis – albeit limited – comparing crossing complexity and knot complexity.

Reconsider the distribution of crossing complexity for equilibrium, intermediate, and fractal-like snapshots (figure R4a-c; figure 4d-e maintext). Roughly speaking, each distribution corresponds to the spectrum of inter-chain entanglements (recall that each snapshot in this analysis had two curves each). The message is that fractal-like configurations mitigate entanglement between two curves (chromosomes).

Next consider the spectrum of knots in equilibrium, intermediate, and fractal-like snapshots (figure R4d-f). We picked one of two chains (at random) in each of the 3x800 snapshots (we maintain that knot complexity does not generalize well to the case of more than one curve). We compute the knot determinant (Alexander polynomial at -1) for each snapshot. Equilibrium snapshots contain an abundance of knots; fractal-like – and to some extent intermediate – configurations mitigate knot complexity.

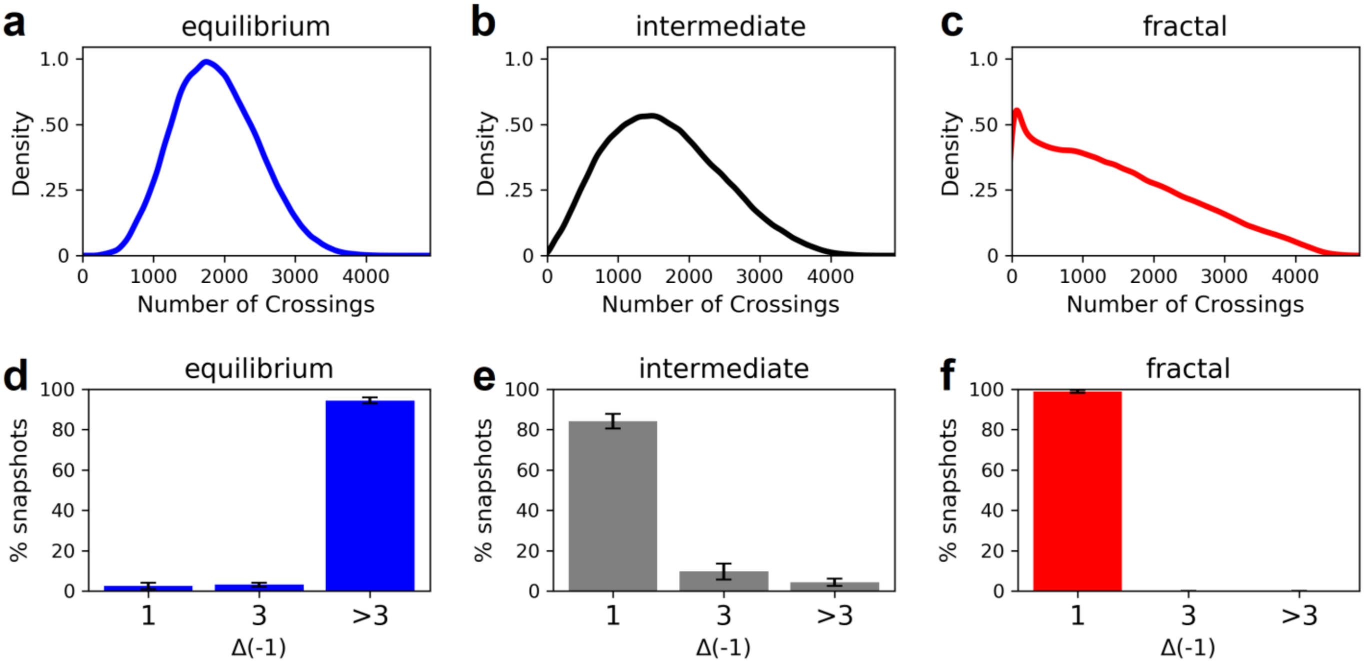

Figure R4 – crossing complexity and knot determinants for ensembles of 800 equilibrium intermediate and fractal-like snapshots. Top row, crossing complexity for ensembles of equilibrium, intermediate and Hilbert-like configurational snapshots. Bottom row, Spectrum of knot determinants (Alexander polynomial at -1) for ensembles of equilibrium, intermediate and Hilbert-like configurational snapshots. Curve closure was sampled 100 times on a on a spere taking the minimum not determinant for each snapshot.

The most appropriate message is not that one metric is better than the other; rather, crossing complexity and knot complexity complement each other. Crossing complexity is useful for quantifying inter-chain (chromosome) entanglement; knot complexity is useful for quantifying intra-chain (chromosome) entanglement.

If further developed this could be an interesting line of analysis. However, the focus of this manuscript is to learn more about crossing complexity. A thorough quantitative comparison of various entanglement metrics is beyond the scope of this work. We revise the second paragraph of the discussion to make this point clear:

> Despite these contributions, crossing complexity remains understudied compared related metrics such as knot complexity (Δ) and surface smoothness (β). Here surface smoothness refers to the monomers of one curve in contact with other curves governed by the exponent β: n_{surf}~N^{β}. Hilbert-like curves are characterized by β = 2/3, knot complexity Δ(-1) = 1, and modal crossing complexity (see results) approximately zero. It is natural to seek a quantitative comparison of these metrics. We hypothesize the correlation is strongest for crossing complexity and surface smoothness; however, a thorough study is beyond the scope of this work.

Finally, we cite previous attempts to quantify entanglement between polymer chains using the Gauss linking number:

> Attempts have been made to quantify entanglement between polymers (chromosomes) with variants of the Gauss linking number (26). Linking number increases with polymer length and density (27, 28).

Honestly, I am missing the message on the S-phase DNA model (Fig. 5): true, there crossing complexity can not distinguish between equilibrium and fractal models, but I think this is just because the model consists of a new chain (say, the red one) which is built to run in parallel with the blue one. Chain crossing is based on local moves so I think any two chains running in parallel (regardless of the details of their global folding in space) should always produce the same result.

First, we want to make sure the reviewer and reader understand the crossing complexity. We have made a small change to clarify that the two curves are completely separated along the axis of each test direction. We are not sure what the reviewer means by "local moves"; suffice it to say each translation separates the curves essentially to infinity. The revised passage regarding crossing complexity clarifies:

> Putative translations are applied over a set of test directions that evenly cover the space S^2 (spherical surface). The two curves are separated – to completion – along the axis of each test direction while counting their mutual crossings (figure 3b).

We also clarify the message regarding S-phase DNA as we segue to these results:

> We hypothesize that the configuration of replicated S-phase DNA influences the number of crossings needed for strand separation. We test this hypothesis in two steps. First, we construct snapshots of S-phase DNA (described below). Then we compare crossing complexity for equilibrium, intermediate, and fractal-like ensembles. The results – which to not support our hypothesis – lead to one of the main conclusions of this study: the

crossing complexity of doubled space filling curves is the same regardless of how they are folded.

Roughly speaking, the implication is that for some states of DNA – particularly S-phase DNA – folding does not affect entanglement. It does not seem trivial to us that any two chains running in parallel (regardless of the details of their global folding in space) should always produce the same result. Therefore, we provide a new geometric argument to rationalize this result. See figure 10 and its associated response to comments from reviewer one. The geometric argument is also found in the revised manuscript.

To summarize, this paper raises several serious concerns which require some substantial reply by the authors should they wish to resubmit their work to Plos One.

We thank the reviewer for the constructive feedback, the message of the paper has truly improved.
