# Supplementary material for: Crossing complexity of space-filling curves reveals entanglement of S-phase DNA
Source: PLoS One. 2020 Aug 31;15(8):e0238322. doi: 10.1371/journal.pone.0238322 (PMC7458320; doi:10.1371/journal.pone.0238322)
Supplement: Attachment — Submitted filename: Response to Reviewers.pdf [file pone.0238322.s005.pdf]

VCOM
Edward Via College of Osteopathic Medicine
VIRGINIA • CAROLINAS
AUBURN

Primary Care Research Network
Center for Bioinformatics and Genetics
Nicholas Kinney, PhD
2265 Kraft Drive
Blacksburg, Virginia 24060
Phone: 301.338.1181
Email: nkinney@vcom.vt.edu

August 8, 2020

Dr. Miguel Angel Sanchez Granero
Academic Editor of PLOS ONE
RE: POINT BY POINT RESPONSE TO REVIEWERS (SECOND REVISION)

Dear Dr. Granero:

In the pages that follow we respond point-by-point to each of the reviewer comments concerning our manuscript: "**CROSSING COMPLEXITY OF SPACE FILLING CURVES REVEALS ENTANGLEMENT OF S-PHASE DNA**". The manuscript is a minor revision that address several additional oversights pointed out by reviewer one.

We are excited to share our revised manuscript with the scientific community and look forward to hearing back. There are no related articles published or under consideration. If there are any specific questions that need a conversation, my cell is 301 338-1181.

Sincerely,

Dr. Nicholas Kinney
Primary Care Research Network and the Center for Bioinformatics and Genetics, VCOM
Professor of Biomedicine, VCOM

Reviewer #1

Reviewer #1: Authors in many places made a good job responding to the critical remarks from the report. but in some places their presentation still needs polishing.

We thank the reviewer for the helpful comments.

Authors start defining space-filling curves as "configurations with fractal dimension equal to the embedding space". Apart from clumsy English (dimension equal ... space), this definition is wrong. For instance, one can easily imagine a space filling curve in 2D with fractal dimension 1 -- a tight spiral starting from zero and filling a circle.

This passage was an oversight. We were probably referring to classical curves such as Peano, Hilbert, and Moore space filling curves. Obviously, our work considers equilibrium and intermediate configurations that render this passage incorrect. In fact, it may be possible to relate fractal dimension to crossing complexity. We now elude to this possibility in the discussion where we also suggest a relationship between crossing complexity and surface smoothness (β). It would be interesting to see a thorough comparison of these measurements; however, that will have to wait for another study.

Some statements are also unclear, such as, e.g., "the 3D distance between any two monomers (R) cannot exceed their 2D separation along the polymer backbone" For is 2D separation?

This was another oversight. The sentence should read as follows:

> For example, the 3D distance between any two monomers (*R*) cannot exceed their 1D separation along the polymer backbone (*s*).

Authors continue "In polymer physics this constraint is expressed as an inequality (R <= s)..." and accompany this with reference to de Gennes. I don't remember such statement in de Gennes book; if it is there, authors should be more specific in their referencing.

The inequality ($R <= s$) is implied but never explicitly stated. In retrospect, it is implicit in any discussion of polymers which makes a specific citation inappropriate. We have tweaked the wording of the sentence and removed the citation. The de Gennes book is still cited at the end of the paragraph.

Around line 250, authors claim that ergodic algorithm should produce torsion-free curves. Why? It is not obvious to me.

Actually, the statement we make – that chain configurations are torsion free – is inaccurate. Just look at figure 7 panel b and c. Most configurations have a degree of torsion. So what do we expect in an unbiased sample of configurations? Consider a hypothetical lattice configuration with a degree of

right-handed torsion. There must be a “mirror image” configuration with the same degree of left-handed torsion. In fact, for every configuration with right-handed torsion there should be a configuration with equal left-handed torsion. Thus, we expect the distribution of torsion in an unbiased ensemble to be symmetric (not necessarily bell-shaped) around zero. We have appended this argument to the passage around line 250.

There is a chapter called "STATISTICS OF SPACE FILLING CURVES SUGGEST THEY ARE EGODIC" Curves are ergodic? What does it mean? I never heard of ergodicity viewed as a property of a curve.

This was a poor choice of words. We change the section heading to “EGODIC SAMPLING OF LATTICE SPACE FILLING CURVES”
